# Bovine Milk Polar Lipids: Lipidomics Advances and Functional Perspectives

**DOI:** 10.3390/foods15020256

**Published:** 2026-01-10

**Authors:** Giulia Fappani, Zhiqian Liu, Simone Rochfort, Gabriele Rocchetti

**Affiliations:** 1 Department of Animal Science, Food and Nutrition (DiANA), Università Cattolica del Sacro Cuore, Via Emilia Parmense 84, 29122 Piacenza, Italy; giulia.fappani@unicatt.it; 2 Department of Energy, Environment and Climate Action, AgriBio, Centre for AgriBioscience, La Trobe University, Bundoora, VIC 3083, Australia; zhiqian.liu@agriculture.vic.gov.au (Z.L.); simone.rochfort@agriculture.vic.gov.au (S.R.); 3 School of Applied Systems Biology, La Trobe University, Bundoora, VIC 3083, Australia

**Keywords:** milk lipidomics, sphingolipids, phospholipids, LC-MS, dairy quality, bioactive lipids, multi-omics

## Abstract

Bovine milk is a complex biological fluid whose lipid fraction plays essential roles in nutrition, processing, and product quality. While conventional analyses have traditionally focused on total fat content and fatty acid composition, recent advances in liquid chromatography–mass spectrometry (LC–MS) have unveiled the molecular diversity of polar lipids, particularly phospholipids and sphingolipids. These compounds, largely associated with the milk fat globule membrane (MFGM), include key molecular species such as phosphatidylcholine (PC), phosphatidylethanolamine (PE), sphingomyelin (SM), ceramides (Cer), and lysophospholipids, which collectively contribute to emulsion stability, flavor development, and bioactive functionality. This review summarizes current progress in the determination of sphingolipids and phospholipids in bovine milk, with a specific focus on analytical strategies enabling their accurate detection, identification, and quantification. We discuss how advanced LC–MS platforms have been applied to investigate factors shaping the milk polar lipidome, including lactation stage, animal diet, metabolic and inflammatory stress, and technological processing. Accumulating evidence indicates that specific lipid species and ratios, such as PC/PE balance, SM and ceramide profiles, and Lyso-PC enrichment, act as sensitive molecular indicators of membrane integrity, oxidative status, heat stress, and processing history. From an applied perspective, these lipidomic markers hold strong potential for dairy quality control, shelf-life assessment, and authenticity verification. Overall, advanced lipidomics provides a robust analytical framework to translate molecular-level lipid signatures into actionable tools for monitoring cow health, technological performance, and the nutritional valorization of bovine milk.

## 1. Introduction

Milk is a highly complex and dynamic biological fluid that provides essential nutrients, bioactive molecules, and structural components supporting growth and health [1,2,3,4]. Beyond its nutritional value, milk represents a biochemical system finely tuned by genetics, physiology, and environment, reflecting the animal’s metabolic status and production conditions [5,6,7]. Among milk constituents, lipids are of particular importance, influencing energy density, sensory properties, and technological behavior. However, the milk lipid fraction also contains a rich diversity of polar lipids, notably phospholipids and sphingolipids, that perform structural and signaling functions and are increasingly regarded as key indicators of milk quality and functionality [8,9].

Traditionally, milk lipid analysis has been limited to the quantification of total fat and the profiling of fatty acids, which have served as proxies for nutritional evaluation and authenticity control [10]. However, these approaches overlook the molecular complexity of the milk fat globule membrane (MFGM), a tri-layered structure surrounding lipid droplets that forms the interface between the aqueous and lipid phases. Although the MFGM represents less than 2% of total milk fat, it is highly enriched in phospholipids and sphingolipids, mainly phosphatidylcholine (PC), phosphatidylethanolamine (PE), phosphatidylserine (PS), phosphatidylinositol (PI), lysophospholipids, and sphingomyelin (SM) [11]. Beyond their structural role in emulsion stability and creaming behavior, these polar lipids exert relevant biological functions linked to intestinal health, cognitive development, and immune modulation [12,13,14]. For a long time, the detailed characterization of these lipid species remained analytically challenging, as conventional chromatographic and spectroscopic techniques lacked the resolution required to discriminate structurally similar compounds, resulting in an incomplete understanding of MFGM composition and dynamics [15]. Recent advances in high-resolution liquid chromatography–mass spectrometry (LC–MS) have markedly advanced milk lipid analysis. Modern platforms, including hybrid quadrupole–time-of-flight and Orbitrap systems coupled with electrospray ionization and tandem MS fragmentation, now enable the sensitive and accurate detection of hundreds of lipid molecular species [16]. The incorporation of complementary strategies such as ion mobility spectrometry, Paternò–Büchi reactions, and ultraviolet photodissociation has further improved structural elucidation, consolidating milk lipidomics as a rapidly evolving field at the interface of analytical chemistry, nutrition, and dairy technology [17,18].

In bovine milk, lipidomics has revealed that the polar lipidome is highly responsive to biological and environmental drivers, including lactation stage, parity, diet composition, heat stress, and oxidative status [19]. In parallel, technological treatments such as pasteurization, homogenization, and storage have been shown to significantly reshape the composition and organization of MFGM lipids, with direct consequences for shelf-life, sensory properties, and digestibility [4]. These observations support sphingolipidomics and phospholipidomics as particularly informative approaches for milk quality, freshness, and safety assessment across the dairy supply chain [20,21,22]. Importantly, the integration of lipidomics with other omics disciplines, including metabolomics, proteomics, and metagenomics, is enabling a more holistic understanding of milk biology, linking lipid metabolism to animal physiology and product functionality, and fostering the development of data-driven quality indicators and predictive models [4]. Despite this potential, several analytical and interpretative challenges remain. The extraction and quantification of minor polar lipids from complex matrices such as milk require standardized and reproducible protocols, as these compounds are susceptible to oxidation, surface adsorption, and ion suppression effects [23]. Moreover, the structural diversity of sphingolipids, arising from multiple sphingoid bases and acyl chain variants, necessitates advanced data-processing workflows and well-curated databases to ensure confident annotation [24]. Addressing these challenges is a prerequisite for the routine implementation of lipidomics as a robust and reliable tool for milk quality and safety assessment.

This review provides a comprehensive overview of recent advances in the sphingolipidomics and phospholipidomics of bovine milk, with emphasis on analytical developments, compositional insights, and quality-related applications. By focusing exclusively on milk rather than processed dairy products, it highlights how polar lipid profiling can serve as both a window into animal physiology and a diagnostic platform for product quality. Within the broader framework of emerging detection technologies, lipidomics stands out as a pivotal approach for ensuring transparency, authenticity, and functionality in the modern dairy sector. Studies included in this review were identified through systematic searches in Scopus, Web of Science, and PubMed, using combinations of the terms ‘milk lipidomics’, ‘phospholipids’, ‘sphingolipids’, ‘high-resolution mass spectrometry’, and ‘bovine milk’. Only peer-reviewed studies focusing on bovine milk polar-lipid research and reporting LC–MS-based lipidomic workflows were considered.

## 2. Polar Lipids in Bovine Milk: Composition and Functional Roles

The lipid fraction of bovine milk is composed of a remarkably diverse set of molecular species, ranging from neutral lipids, mainly triacylglycerols (TAGs, accounting for more than 95% of total fat), to a variety of minor components including diacylglycerols, cholesterol esters, phospholipids, and sphingolipids. While TAGs serve primarily as an energy reservoir and influence rheological and sensory properties, minor polar lipids, located mainly in the milk fat globule membrane (MFGM), are central to the structural organization and biological functionality of milk [25,26]. These polar lipids represent approximately 0.3–1% of total milk lipids but exert disproportionate effects on the physical stability, technological behavior, and health-related attributes of milk [11,27]. A schematic representation of the structure of MFGM is reported in f1.

### 2.1. Phospholipids

Phospholipids (PLs) constitute the major polar lipid class in bovine milk. They are amphiphilic molecules comprising a glycerol backbone esterified with two fatty acids and a phosphate-containing head group. Many species of PLs exist within each class, depending on the variety of the two fatty acids that compose the molecule. The primary subclasses include (PC), (PE), (PS), (PI), and phosphatidic acid (PA), along with their corresponding lysophospholipids (LPC, LPE) that originate from partial hydrolysis of PC and PE [28]. Among these, PC and PE typically account for 60–70% of total PLs, followed by SM (which is often classified with sphingolipids but structurally bridges both classes). The fatty acid composition of milk PLs is characterized by a high proportion of saturated and monounsaturated chains, such as palmitic (C16:0), stearic (C18:0), and oleic (C18:1) acids, though polyunsaturated fatty acids (PUFA) including linoleic (C18:2) and arachidonic (C20:4) acids. In addition, odd- and branched-chain fatty acids are also present in smaller amounts [29,30]. The diversity of fatty acyl chains contributes to membrane fluidity and influences the physicochemical properties of milk fat globules. The PL content in the final dairy product is related to the type of technological process applied, and may cause fractionation or separation of fat globules, but also disruption of the membrane [31,32]. Functionally, PLs are essential for the emulsifying capacity of milk, stabilizing the fat-water interface and preventing coalescence during storage and processing. Moreover, PLs are precursors for bioactive mediators such as lysophosphatidylcholine and platelet-activating factor, linking milk composition with metabolic and immunological functions in the neonate. In addition, alterations in the phospholipid structure of milk (e.g., changes in FA and/or double bond location), could be potential biomarkers for inflammatory or metabolic disease in cows [30].

### 2.2. Sphingolipids

Sphingolipids (SP) are another crucial class of polar lipids in bovine milk, structurally distinct from glycerophospholipids. They are based on a sphingoid backbone (typically sphingosine or dihydrosphingosine) amide-linked to a fatty acid (generally a saturated fatty acid), forming ceramides that serve as precursors for more complex derivatives such as sphingomyelin (SM) and glycosphingolipids (e.g., glucosylceramide, lactosylceramide, and gangliosides). Among these, SM is by far the dominant sphingolipid in bovine milk, accounting for approximately 25–30% of total MFGM polar lipids. It consists of a phosphorylcholine head group linked to a ceramide [33]. Sphingolipids perform multiple roles. Structurally, they contribute to the rigidity and organization of the MFGM through their high melting points and ability to form ordered lipid domains [27]. Biologically, they participate in cell signaling, apoptosis, and inflammatory regulation, but they also act as receptors for some growth factors, hormones and toxins [34]. In human nutrition, milk-derived sphingomyelin and gangliosides have attracted considerable attention for their potential neurodevelopmental and gut-barrier functions [35]. In bovine milk, sphingolipid levels are influenced by physiological factors such as animal diet, stage of lactation, parity, udder health, and metabolic stress, as well as by thermal treatments that can promote degradation or isomerization of labile species [36,37,38].

### 2.3. The Milk Fat Globule Membrane as a Lipid Reservoir

**Figure 1:**
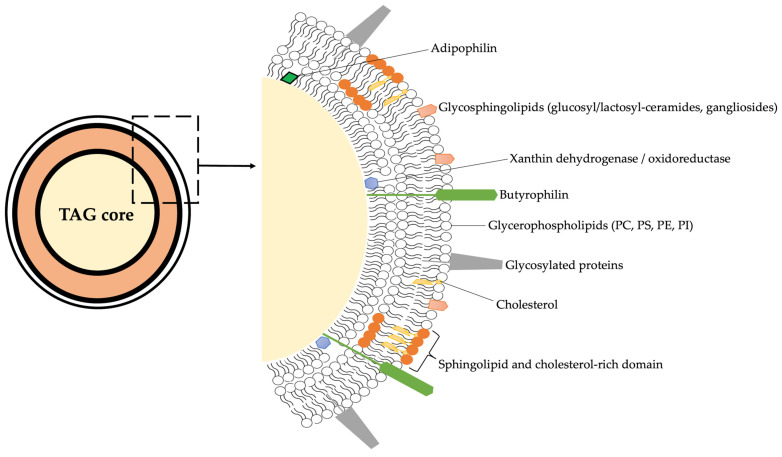
Schematic overview on the structure of MFGM considering all the main chemical components. PC = phosphatidylcholine; PS = phosphatidylserine; PE = phosphatidylethanolamine; PI = phosphatidylinositol; TAG = triacylglycerol.

The milk fat globule membrane (MFGM) acts as a natural emulsifier and barrier, enclosing lipid droplets secreted by mammary epithelial cells. Its tri-layered structure includes a cytoplasmic inner monolayer derived from the endoplasmic reticulum and an outer bilayer originating from the apical plasma membrane [25]. This complex organization explains the enrichment of phospholipids and sphingolipids within the MFGM and their tight association with membrane proteins such as butyrophilin, xanthine oxidase, and adipophilin (Figure 1 and Table 1).

The MFGM composition is dynamic and responds to both biological and technological factors. For example, pasteurization and homogenization can alter membrane integrity, induce lipid-protein rearrangements and lipidome remodeling, potentially affecting the distribution and bioavailability of polar lipids [4]. Recent LC-MS-based lipidomic studies have revealed that the MFGM lipidome is highly variable among cows, farms, and even individual milkings, reflecting complex interactions between genetics, diet, and environmental stressors. Such sensitivity makes the polar lipid fraction an informative biomarker reservoir for assessing milk quality, freshness, and animal physiological status. Moreover, given their amphiphilic nature, MFGM lipids are key determinants of milk foamability, heat stability, and mouthfeel-parameters closely linked to consumer perception and industrial processing performance [39]. Beyond their technological roles, milk PLs and sphingolipids have drawn attention to their potential health benefits. Several studies indicate that milk-derived phospholipids can modulate lipid metabolism, reduce cholesterol absorption, and exert anti-inflammatory effects. Sphingomyelin and its metabolites, ceramide and sphingosine, are involved in maintaining intestinal epithelial integrity and regulating immune responses [38]. These findings have stimulated interest in enriching dairy-based ingredients (e.g., MFGM concentrates) for functional nutrition and infant formula development [3]. From an analytical perspective, the quantification of these bioactive lipids in bovine milk requires high sensitivity and structural resolution, achievable only through modern LC-MS lipidomic platforms.

## 3. Analytical Advances in Milk Lipidomics

**Figure 2:**
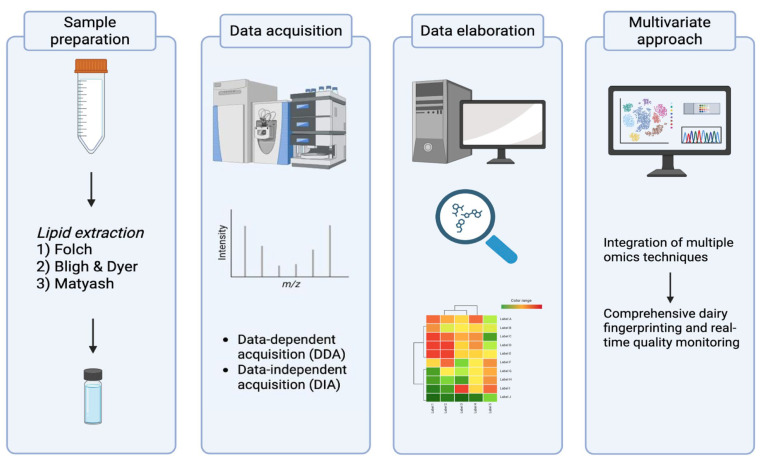
Graphical overview of the lipidomics workflow applied to milk samples.

The detailed characterization of phospholipids and sphingolipids in bovine milk requires analytical workflows capable of resolving complex molecular structures at trace concentrations within a highly heterogeneous matrix [18,40]. The polar lipid fraction, although minor, plays crucial roles in milk functionality and bioactivity, and its accurate quantification is essential for understanding how technological and physiological factors influence milk quality. Recent developments in liquid chromatography–mass spectrometry (LC–MS) have transformed this field, providing unprecedented sensitivity, selectivity, and structural resolution. However, lipidomic analysis of milk remains challenging due to the high fat content, co-extraction of proteins and sugars, and wide polarity range of lipid species. Optimized workflows involving efficient extraction, chromatographic separation, and advanced MS detection are therefore critical. An overview of the main steps of a lipidomics-based workflow is summarized in Figure 2.

### 3.1. Sample Preparation and Lipid Extraction

Efficient and reproducible extraction of polar lipids from milk is a key prerequisite for reliable lipidomic analysis. The complexity of the milk matrix, comprising emulsified lipids, casein micelles, and whey proteins, necessitates a strategy that maximizes lipid recovery while minimizing matrix effects. The Folch and Bligh & Dyer methods, based on chloroform–methanol partitioning, respectively, remain the most widely applied protocols for milk lipid extraction [41,42]. These biphasic systems, (1:2, *v*/*v*) or (2:1, *v*/*v*) MeOH:CHCl_3_ for the Folch and Bligh & Dyer, respectively, efficiently extract both neutral and polar lipids, though the choice of solvent ratio can markedly influence the recovery of phospholipids and sphingolipids. Modified versions, such as MTBE-based extractions, offer improved phase separation and reduced solvent toxicity. In particular, the Matyash method (MTBE/methanol/water) has gained popularity in lipidomics for its ability to extract a wide polarity range and yield cleaner lipid phases suitable for LC-MS. In addition, a single-phase method for lipid extraction from milk has also been reported, offering a rapid and reproducible alternative to some other sample preparation techniques. Given the low abundance of polar lipids in milk (typically < 1% of total lipids), pre-concentration or clean-up steps are sometimes necessary [43]. Solid-phase extraction (SPE) using aminopropyl or silica cartridges allows partial enrichment of PLs and sphingolipids, facilitating downstream analysis. However, excessive manipulation can lead to selective losses, particularly for lysophospholipids and labile sphingolipid intermediates [44]. The use of internal standards, preferably isotopically labeled analogs for each lipid class, is essential to correct for extraction variability and matrix effects during ionization [45,46]. Another critical consideration is sample freshness and storage. Polar lipids are sensitive to oxidation and enzymatic degradation; thus, immediate freezing at −80 °C and avoidance of multiple thawing cycles are recommended. Milk samples collected under varying physiological or technological conditions (e.g., heat-treated, homogenized, or subjected to oxidative stress) as previously reported [20,47,48], require validation of extraction efficiency to ensure accurate comparisons across treatments.

### 3.2. Chromatographic Separation Strategies

The structural diversity of milk PLs and sphingolipids demands chromatographic systems capable of separating molecular species differing in headgroup polarity, acyl chain length, or degree of unsaturation. Liquid chromatography (LC) is the preferred front-end technique for lipidomics, providing both retention-based selectivity and compatibility with MS detection [45,49,50]. Reverse-phase LC (RP-LC), typically employing C18 or C8 columns, separates lipids primarily by hydrophobic interactions and acyl chain characteristics. It offers excellent resolution of isobaric and analogous species differing in fatty acid composition and is thus suitable for profiling of molecular species within the same lipid class (e.g., PC 34:1 vs. PC 34:2). However, RP-LC provides limited separation between lipid classes with different polar headgroups [51]. In contrast, hydrophilic interaction chromatography (HILIC) provides complementary selectivity, resolving lipid classes according to headgroup polarity. HILIC has proven particularly effective for separating PC, PE, PS, and SM in milk, often achieving class-based grouping with sharp peak shapes. Moreover, HILIC is compatible with both positive and negative electrospray ionization (ESI), enabling dual-polarity detection of lipid subclasses [52,53]. Recent studies have also explored supercritical fluid chromatography (SFC) as a high-throughput alternative for complex lipid mixtures. SFC offers faster separations, reduced solvent consumption, and improved resolution for nonpolar and amphiphilic species, though method optimization for milk matrices remains ongoing [54,55]. Gradient optimization, column temperature, and mobile phase modifiers (e.g., ammonium formate, ammonium acetate) significantly affect ionization efficiency and reproducibility. The choice of chromatographic mode is ultimately determined by the analytical objective; particularly, RP-LC is favored for detailed molecular profiling and relative quantification within classes; HILIC is ideal for class-level quantification and discovery-based lipidomics; SFC or multi-dimensional LC approaches are emerging for comprehensive lipidome coverage [56].

### 3.3. Mass Spectrometry Developments and Data Acquisition Strategies

Mass spectrometry (MS) represents the core of lipidomic analysis, offering unparalleled sensitivity and specificity for structural elucidation [57]. Recent advances in high-resolution mass spectrometry (HRMS) have enabled the detection of hundreds of PL and sphingolipid species in milk, providing detailed insight into lipid metabolism and quality attributes. Electrospray ionization (ESI) remains the most common ionization technique due to its soft ionization and compatibility with LC solvents [58]. PLs PC, PE generally ionize efficiently in positive mode ([M + H]^+^), PI in negative mode ([M − H]^−^), whereas PS can be detected in both positive and negative mode [16]. Sphingomyelins, due to their quaternary ammonium group, typically exhibit strong signals in positive mode [59]. For structural elucidation, tandem mass spectrometry (MS/MS) is indispensable. Fragmentation patterns reveal headgroup-specific ions (e.g., *m*/*z* 184 for PC/SM in positive mode) and acyl-chain fragments that allow assignment of fatty acid composition [60]. The use of data-dependent acquisition (DDA) facilitates identification of unknown lipids based on precursor ion selection, whereas data-independent acquisition (DIA) and SWATH-MS approaches improve reproducibility and comprehensiveness for quantitative workflows [61]. Targeted methods employing triple quadrupole (QQQ) or quadrupole-linear ion trap (QTrap) instruments enable high-sensitivity quantification through multiple reaction monitoring (MRM), making them ideal for biomarker validation or routine quality monitoring [62]. For discovery-oriented studies, hybrid Orbitrap and QTOF systems offer sub-ppm mass accuracy and high resolving power, crucial for distinguishing isobaric species in complex milk extracts [63,64]. Internal standardization and normalization are essential for quantitative reliability. Class-specific standards (e.g., labeled standards from Avanti Lipids) enable correction for matrix effects and ionization differences, while total ion current (TIC) or lipid-class normalization can be applied in untargeted workflows. Furthermore, the use of lipid-specific databases and software tools, such as LipidSearch (version 5.0), LipidBlast database, and MS-DIAL (version 4.90), facilitates accurate annotation and molecular assignment, although manual verification remains critical for avoiding misidentifications, especially among sphingolipids with similar backbone masses [23,61,65,66,67]. A major analytical challenge in milk lipidomics is the presence of isobaric and isomeric lipid species, which share identical nominal masses and often co-elute chromatographically [68]. This complexity hampers accurate annotation and quantification, particularly for PLs differing only in acyl chain position, unsaturation geometry, or sphingoid base structure. Recent advances in ion mobility spectrometry coupled to mass spectrometry (IMS–MS) have provided an additional dimension of molecular separation based on ion shape, size, and charge. By measuring the collision cross section (CCS) of ions as they drift through a neutral gas under an electric field, IMS discriminates lipid isomers that cannot be resolved by mass or retention time alone [69]. When combined with LC–MS, this multidimensional approach (LC–IMS–MS) significantly enhances the structural resolution of complex lipid mixtures in milk [70]. For example, it enables the differentiation between sn-positional isomers of PC or ceramide species with distinct hydroxylation patterns, offering a more accurate representation of lipid diversity. Moreover, CCS values are reproducible and can serve as orthogonal identifiers in lipid databases, complementing MS/MS fragmentation data. The integration of trapped ion mobility (TIMS) and cyclic ion mobility (cIMS) instruments has further improved sensitivity and speed, opening new avenues for high-throughput, structure-resolved lipidomics of dairy matrices [70,71,72]. Despite remarkable analytical progress, lipidomics of bovine milk still face challenges related to method harmonization, quantitative accuracy, and inter-laboratory reproducibility. Differences in extraction protocols, chromatographic conditions, and ionization settings often lead to variable results, complicating comparisons among studies. Efforts toward standardized workflows, quality control samples, and reference materials for PLs and sphingolipids are therefore essential. The establishment of lipidomic proficiency testing schemes, similar to those in metabolomics, would further enhance data reliability and comparability [17,73]. Ultimately, the integration of robust LC-MS methods with bioinformatics pipelines and machine-learning algorithms holds great promise for next-generation milk lipidomics, capable of translating complex molecular fingerprints into actionable indicators of milk quality, authenticity, and safety.

## 4. Bioactive and Functional Lipid Biomarkers in Bovine Milk

Advances in lipidomics have unveiled a rich diversity of PL and sphingolipid species in bovine milk that not only shape its nutritional and technological properties but also act as biomolecular indicators of metabolic status, animal health, and processing impact. These lipids, once considered minor components of the milk fat globule membrane (MFGM), are now recognized as sensitive biomarkers reflecting physiological and environmental perturbations throughout the dairy supply chain. Their dynamic modulation provides valuable insights into both on-farm conditions and post-harvest processing, bridging biological function with quality assurance. An overview of the most interesting polar lipid biomarkers (from lipidomics-based studies) as related with both animal-, stress-, and technological-factors can be found in Table 2.

### 4.1. Animal-Related Determinants and Physiological Stress

At the animal level, the composition of milk PLs and sphingolipids is tightly regulated by lactation stage, parity, metabolic status, and inflammatory condition [38,96,97]. The genetic background of the cow also contributes to polar-lipid architecture, largely through effects on MFG size and secretion pathways [98]. These effects are increasingly evident when comparing breeds or species, reflecting evolutionary differences in membrane biosynthesis and lipid trafficking. During early lactation, intense lipid mobilization and oxidative stress can alter the (PC/PE) ratio, often interpreted as a marker of membrane stress. (SM) and ceramide (Cer) species also vary with mammary inflammation and subclinical mastitis, consistent with the role of ceramide-mediated signaling in apoptosis and immune activation. Changes in lysophospholipids (Lyso-PC, Lyso-PE) reflect phospholipase activity and oxidative processes linked to metabolic imbalance or stress [99]. Likewise, (PS) and (PI) shifts have been associated with epithelial turnover and immune modulation, particularly under heat stress or negative energy balance. Diet further modulates polar-lipid composition: supplementation with unsaturated fats, antioxidants, or choline donors increases long-chain SM and PE species, improving membrane fluidity and oxidative stability. Recent data also show that specific feed-derived bioactives can enhance phospholipid synthesis and antioxidant signatures in milk [93]. Becchi et al. [95] recently applied an integrated UHPLC-HRMS metabolomics and IM-HRMS lipidomics approach to characterize Parmigiano Reggiano Protected Designation of Origin (PDO) milk, highlighting polar lipids as sensitive markers of feeding system and milking time. While forage type mainly influenced the small-molecule metabolome, lipidomics specifically revealed changes in SM and SP-associated lipid assemblies, reflecting modulation of the MFGM. In particular, SM emerged as key contributors to differences between morning and evening milk, likely linked to feeding patterns and circadian metabolism. A recent lipidomics comparison of bovine and donkey milk identified >200 polar lipids, with 150 species differing between the two matrices, mainly related to glycerophospholipid and sphingolipid metabolism [100]. Similarly, Fan and co-authors [74] reported pronounced species-specific MFGM lipid signatures across yak, buffalo, and Holstein milk, detecting 1748 lipid species and uniquely identifying methylated phospholipids such as BisMePA and MePC. Shared differences mapped to glycerophospholipid and PUFA metabolism pathways, highlighting conserved regulatory axes and species-adapted milk functions. Heat stress represents another major determinant. Acute heat stress with a temperature-humidity index (THI) ≈ 84 led to a decrease in short- and medium-chain TAGs and a reduction in polar lipid classes including PE, PS, PC, Lyso-PC, and GluCer [76]. Lyso-PC emerged as a discriminatory marker for heat-tolerant vs. susceptible animals, supporting its potential as a dairy heat-stress biomarker. In contrast, mild short-term heat stress did not substantially impact polar lipids, underscoring the threshold-dependent nature of lipidome adaptations [101]. Beyond temperature, other metabolic challenges such as ketosis also reshape milk sphingolipid profiles, including increases in Cer and SM species linked to adipose mobilization and oxidative stress [90]. Ceramides are increasingly recognized as metabolic mediators in dairy cows. As discussed by McFadden and Rico [102] postpartum lipolysis drives hepatic ceramide synthesis, contributing to insulin resistance and nutrient prioritization. Palmitic acid supplementation can modulate Cer pools, offering a nutritional lever to influence milk yield and metabolic resilience. Taken together, these findings reinforce that milk polar lipids reflect nutrient partitioning, immune tone, and thermal and metabolic load—positioning milk lipidomics as a promising non-invasive tool for precision monitoring of cow health and welfare.

### 4.2. Technological and Processing-Related Modifications

Once milk leaves the udder, its lipidomic profile continues to evolve under the influence of technological treatments. The MFGM is particularly sensitive to thermal, mechanical, and oxidative stresses, and these processing steps can markedly reshape the abundance and structural integrity of PLs and SP. Pasteurization and homogenization are well-established drivers of MFGM remodeling, promoting redistribution and partial hydrolysis of key PL classes [75]. Heat treatment typically reduces SM and PC content through coalescence and lipase-mediated hydrolysis, while favoring the accumulation of lysophospholipids (e.g., Lyso-PC, Lyso-PE) generated by enzymatic or thermal breakdown [50]. Homogenization, by fragmenting fat globules and exposing membrane components, enhances their interaction with casein micelles and whey proteins, ultimately altering PL surface distribution and potentially compromising bioaccessibility [103].

Ultra-high-temperature (UHT) processing and prolonged storage further accelerate oxidative reactions involving unsaturated PLs, resulting in oxidized PC and PE species that can serve as molecular indicators of heat load and storage stress. In a comprehensive UHPLC-Triple-TOF lipidomic survey, Zhang et al. [50] identified 788 lipids across 29 classes and showed that UHT treatment (135 °C, 4 s) caused more pronounced PL degradation than standard pasteurization (72–85 °C) or extended-shelf-life treatment (121 °C, 15 s), confirming the hierarchy of thermal severity. Emerging evidence also points to technologically resilient SM species (e.g., d18:1/24:0) that remain detectable after harsh processing, while decreases in PS and PI species, and shifts in the SM:Cer ratio, can reveal the extent of membrane hydrolysis and potential biofunctional loss. A recent work by Yan et al. [23] provided mechanistic insight into oxidation dynamics in pasteurized milk, demonstrating that lipid oxidation proceeds post-processing and peaks around day six of refrigeration. Importantly, glycerophospholipids on the inner MFGM leaflet showed greater re-esterification capacity than those on the outer leaflet, while internal glycerides served as the primary oxidation substrates. These findings illuminate how post-processing lipid turnover contributes to quality decay and highlight potential targets for shelf-life optimization.

Beyond heat, other processing strategies also influence polar lipids. Huang et al. [75] showed that pasteurization primarily reduced SM and PC located in the outer bilayer, whereas UHT processing additionally depleted inner-leaflet PI and PE, confirming a leaflet-specific vulnerability of MFGM phospholipids. Similarly, Mou et al. [20] reported that boiling and freezing significantly decreased total PL content and lysophospholipids, with frozen milk retaining only ~70% of its original PL fraction. Fermentation-based transformations also deserve mention: Gao et al. [81] observed significant reductions in PC and PE subclasses during yogurt production, mediated by microbial phospholipases and membrane remodeling. Together, these findings reinforce that both thermal and non-thermal technologies contribute distinctly to the remodeling of the milk polar lipidome. The development of standardized LC-MS fingerprints that track this molecular erosion provides new opportunities for quality control, authentication, and shelf-life prediction. Coupling untargeted profiling with multivariate approaches allows discrimination between raw, pasteurized, and UHT milk based on subtle shifts in phospho- and sphingolipid species, supporting the emergence of lipidomics as a key analytical tool for industrial milk authentication and technological monitoring.

### 4.3. Integrative and Predictive Perspectives

The dual sensitivity of milk polar lipids to biological and technological influences positions them as integrative indicators of both farm-level conditions and processing-related quality [98,104,105]. Lipidomic fingerprints can therefore be leveraged to build predictive models for authenticity, health status monitoring, and technological performance, especially when combined with multi-omics data streams such as metabolomics, proteomics, metagenomics, and sensor-based readouts. For instance, Zhao et al. [90] demonstrated that subclinical ketosis profoundly perturbs the milk lipidome, with 753 lipid species quantified across FA, GL, GPL, and SP classes, and notable alterations in PE, PC, PS, Cer, SM, and HexCer species. However, the lack of integrated metabolite–lipid network analysis in that study highlights the need for harmonized multi-omic interpretation to fully uncover metabolic–lipid cross-talk during subclinical metabolic distress. Likewise, Bellassi and co-authors [106] identified specific lysophospholipids (e.g., LysoPE (22:1), LysoPC (10:0)) as hallmarks of fresh forage/hay feeding systems, and further paired lipidomics with rumen metagenomics to reveal that lysophospholipids display the strongest microbe–lipid correlation networks. Such approaches show that lipid species, particularly Lys-PE and Lys-PC, are not only markers of oxidative or enzymatic remodeling but also sentinels of rumen–mammary metabolic interactions.

The utility of polar lipids in monitoring udder health is further confirmed by Ceciliani et al. [77], who reported pronounced changes in TAGs and SMs during subclinical non-*aureus* staphylococci (NAS) intramammary infections, advancing lipidomics as a non-invasive tool for immune surveillance in dairy herds. Similarly, recent observations suggest that while lipidomics offers strong diagnostic signals for metabolic and inflammatory stress, TAG-based metrics may outperform polar-lipid markers in unrelated health challenges such as lameness—indicating that class-specific prioritization may depend on the physiological target [83]. Such nuance underscores the need to contextualize biomarker panels to specific biological endpoints.

Beyond pathology, lipidomics has gained traction in authentication and market protection. Comparative studies across cow, goat, yak, and camel milk, and even plant-based beverages, show consistent discriminative capacity driven by PC, PE, SM, and lyso-PL subclasses [78,84,85]. Machine-learning models applied to these datasets reliably differentiate species and detect adulteration, demonstrating the promise of lipidomics for traceability and fraud prevention. Looking ahead, the key challenge lies in translating discovery-driven lipidomics into validated, actionable biomarker panels and surveillance tools [107]. Establishing reference lipid libraries, harmonized LC-MS workflows, and inter-laboratory calibration schemes will be essential to support reproducible quantification and cross-cohort standardization. Meanwhile, continued attention to bioactive phospho- and sphingolipid mediators, particularly SM, ceramides, PS, and lysophospholipids, will expand opportunities for functional milk valorization, given their roles in gut barrier support, neural development, and inflammation control. By uniting molecular-level lipid signatures with nutritional, physiological, and technological endpoints, milk lipidomics is poised to underpin next-generation dairy quality assessment, enabling integrated, multi-parameter monitoring across the entire production chain.

## 5. Integrating Lipidomics with Other Omics Platforms and Biofluids

The lipid fraction of milk represents only one dimension of a complex biochemical system in which metabolic, proteomic, and microbiological processes are tightly interconnected. The emerging field of multi-omics integration offers unprecedented opportunities to contextualize lipidomic data within broader metabolic networks, improving our understanding of how lipid signatures relate to physiology, quality, and safety of bovine milk. Integration of lipidomics with metabolomics has proven particularly powerful in delineating the systemic responses of dairy cows to diet, stress, and disease. While metabolomics captures small polar metabolites reflecting instantaneous metabolic activity, lipidomics extends this view to membrane dynamics, oxidative status, and energy balance. A previous work by Cabrera et al. [108] exploited a combined omic approach to profile polar metabolites and lipids in bovine, caprine, and ovine milk samples. Particularly, 414 of 587 (71%) polar metabolites and 210 of 233 (87%) lipids were found significantly different between species. Interestingly, a significant seasonal trend was observed in the polar metabolite fraction for bovine, caprine, and ovine milk, thus suggesting a higher susceptibility of polar metabolites to changes within seasons than bovine milk. Interestingly, PLs (such as PC and PE) showed main variations in caprine milk within the season when compared to other samples. A previous work published by Zhao et al. [109] applying an integrated serum metabolomics-lipidomics approach in transition dairy cows has revealed a marked remodeling of sphingolipid metabolism associated with adipose mobilization and metabolic stress in early lactation. In particular, cows experiencing high lipolysis showed elevated circulating ceramide species such as Cer (d20:0/18:0), Cer (d18:1/16:0), and Cer (d18:0/24:0), together with altered acylcarnitines and amino acid profiles, highlighting a tight link between sphingolipid signaling, insulin resistance, and inflammatory responses. These findings underscore the central role of ceramide accumulation in metabolic adaptation around parturition and suggest that similar sphingolipid-driven regulatory mechanisms may extend to milk lipidomic profiles, where ceramides are emerging markers of metabolic status and mammary lipid metabolism.

Combined analyses have revealed, for instance, that alterations in SM and ceramide profiles often coincide with shifts in amino acid and acylcarnitine metabolism during negative energy balance or inflammatory stress [110]. Such cross-domain correlations highlight the co-regulation between lipid signaling and intermediary metabolism, enabling the identification of composite biomarkers with improved diagnostic performance compared to single analytes. In milk specifically, the joint profiling of polar lipids and aqueous metabolites has enabled discrimination between heat-treated, mastitic, and raw milk, suggesting that lipidomic markers can complement classical metabolomic fingerprints in authenticity and quality monitoring. Given that most PLs and sphingolipids are integral components of the milk fat globule membrane (MFGM), their functional interpretation benefits greatly from integration with proteomic data. Proteomic–lipidomic studies have demonstrated that MFGM composition reflects both the secretory activity of mammary epithelial cells and technological disruption during processing [111,112,113]. Correlations between specific membrane proteins (e.g., butyrophilin, xanthine oxidase) and SM/PC ratios have provided insight into membrane stability and susceptibility to oxidative or enzymatic modification. Moreover, proteo-lipidomic mapping of MFGM components across lactation stages has revealed parallel regulation of PL synthesis enzymes and structural membrane proteins, supporting the concept that lipidomics can serve as a functional mirror of mammary cell physiology.

The milk microbiome is increasingly recognized as a factor influencing lipid composition, both directly through lipolytic activity and indirectly via host-microbe interactions [114]. Integration of microbiomics and lipidomics has started to uncover microbial signatures associated with lipid oxidation, lipase activity, and spoilage potential. In fermented dairy matrices, such as raw milk cheeses or yogurt bases, microbial metabolism can drive formation or degradation of sphingoid bases and lysophospholipids, which in turn affect texture, flavor, and potential bioactivity [115,116]. Although still emerging, such multi-omic approaches offer a holistic view of milk as a living matrix, where lipids function as both targets and mediators of biochemical transformation. Beyond biological interpretation, the integration of lipidomics with other omics layers enables data fusion strategies for quality prediction and authenticity testing. Multivariate and machine-learning models combining LC-MS lipidomics, nuclear magnetic resonance (NMR) metabolomics, and spectroscopic data have shown promise for classifying milk by origin, breed, or processing level with high accuracy. In this sense, the inclusion of phospho- and sphingolipid features, often highly discriminant but underutilized, represents a critical step toward comprehensive dairy fingerprinting and real-time quality monitoring.

## 6. Conclusions and Future Perspectives

Advances in high-resolution lipidomics have profoundly expanded our understanding of the molecular landscape of bovine milk, unveiling the intricate diversity of PLs and sphingolipids that underpin its nutritional, technological, and functional properties. These minor yet bioactive lipids have emerged not only as essential structural components of the MFGM) but also as sensitive biomarkers of animal physiology, environmental stress, and processing impact. Their dual biological and technological responsiveness makes them ideal candidates for integrated quality assessment within modern dairy systems. The next frontier lies in translating lipidomic insights into applicable detection tools. Developing standardized extraction protocols, quantitative workflows, and validated reference libraries will be critical to ensure reproducibility and comparability across studies. Furthermore, the incorporation of ion mobility spectrometry and multi-omic data fusion is expected to enhance structural resolution and interpretative depth, enabling a more holistic view of milk composition. Future research should aim to define robust lipid biomarker panels for specific applications, such as early detection of mastitis, verification of thermal treatment, or monitoring of oxidative stability, while also exploring the functional implications of bioactive milk lipids for human health. By bridging analytical innovation with biological meaning, advanced milk lipidomics holds the potential to become a cornerstone of precision dairy science, providing both a molecular lens on animal health and a powerful analytical platform for ensuring the quality, authenticity, and safety of dairy products.

## Figures and Tables

**Table 1:** Overview on the main lipid categories, classes, and subclasses characterizing the milk fat globule membrane. Lipid classes and subclasses were assigned according to the classification provided by the MS-DIAL software (version 4.90).

Category	Main Class	Lipid Subclass	Structure (Example)
Glycerolipids (GL)	Triradylglycerols	Triacylglycerol (TG)	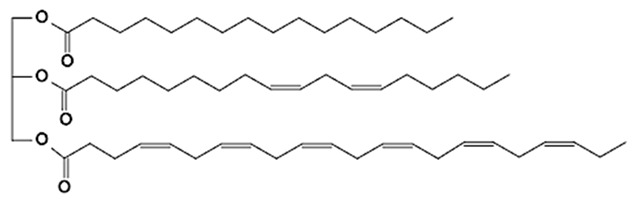
Sphingolipids (SP)	Ceramides	Ceramide non-hydroxyfatty acid-sphingosine (Cer-NS)	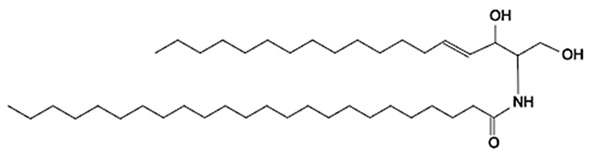
	Acidic glycosphingolipids	Ganglioside GM3 (GM3)	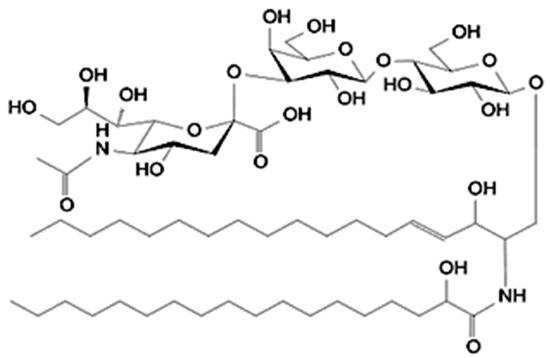
	Phosphosphingolipids	Sphingomyelin (SM)	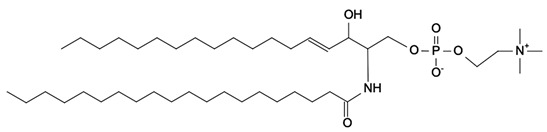
Glycerophospholipids (GP)	Glycerophosphocholines	Phosphatidylcholine (PC)	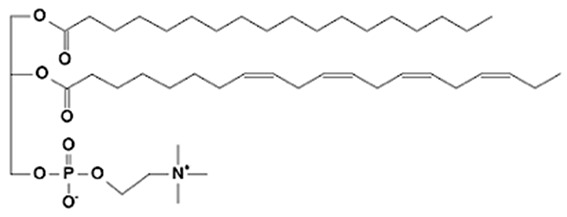
	Glycerophosphoserines	Phosphatidylserine (PS)	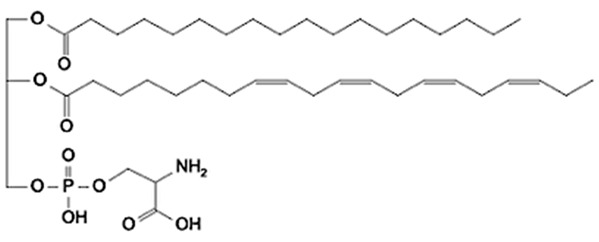
	Glycerophosphoethanolamines	Phosphatidylethanolamine (PE)	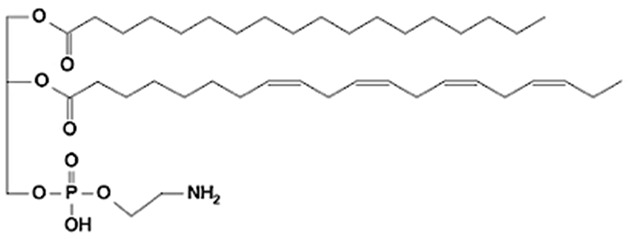
	Glycerophosphoinositols	Phosphatidylinositol (PI)	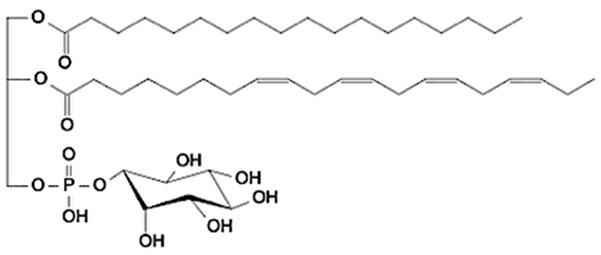
Sterol Lipids (ST)	Sterols	Cholesterol	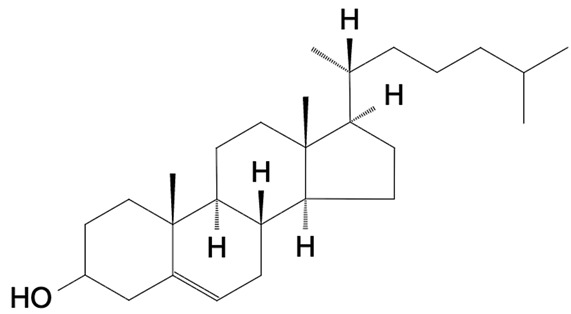

**Table 2:** Recent lipidomics-based workflow dealing with bovine milk and their functionality. The main polar lipid subclasses together with the main findings are comprehensively summarized.

Polar Lipid Subclasses	Lipidomic Workflow	Main Findings	Reference
PC, PE, SM, Hex2Cer, Hex1Cer, PS, PI, methylated PLs	(1) Extraction system: chloroform-methanol solution with a volume ratio of 2:1 (*v*/*v*) (2) Chromatography: BEH C18 1.7 μm (2.1 × 100 mm; Waters) (3) Detection: Untargeted UHPLC-MS	Identified 1748 lipids in MFGM (38 subclasses), including first-time detection of methylated polar PLs (BisMePA, MePC, dMePE). Distinct species-specific polar lipid signatures were found across yak, Holstein, and buffalo milk, driven mainly by glycerophospholipid metabolism.	[74]
PC, PI, PS, SM, PE, PA, LacCer, PG, lyso-PC, lyso-PE, glucosylceramides, PC-P, PE-P, gangliosides, Cer.	(1) Extraction system: chloroform-methanol solution with a volume ratio of 2:1 (*v*/*v*) (2) Chromatography: Luna HILIC column (250 × 4.6 mm, 5 μm, Phenomenex) (3) Detection: Untargeted UHPLC-MS	Identification of 514 polar lipid species (15 classes) in bovine milk, with PC, PE, and SM as the most abundant classes. Detailed structural assignment highlighted the complexity of SP species, providing the first complete reference map of minor milk polar lipids.	[40]
SM, PS, PE, PI, PC, lyso-PE, lyso-PC.	(1) Extraction system: methanol-dichloromethane solution with a volume ratio of 2:0.9 (*v*/*v*) (2) Chromatography: Luna HILIC column (250 × 4.6 mm, 5 μm, Phenomenex) (3) Detection: Untargeted UHPLC-MS	Thermal processing altered MFG structure and MFGM integrity, increasing globule size and fragility while shifting interfacial protein composition. Phospholipidomics revealed 27 differential phospholipid species, with pasteurization mainly reducing outer-leaflet SM and PC, and ultra-pasteurization also depleting PI and PE from the inner membrane layer.	[75]
PS, PI, PE, PC, lyso-PC, SM, LacCer, GluCer	(1) Extraction system: butanol/methanol/chloroform at a 3:5:4 ratio (2) Chromatography: Luna HILIC column (250 × 4.6 mm, 5 µm, Waters) (3) Detection: LTQ-Orbitrap Elite-MS	Acute heat stress altered milk fat composition, reducing TAGs with short-/medium-chain fatty acids and increasing long-chain TAGs, alongside a marked decrease in key polar lipid classes. Lysophosphatidylcholine showed the strongest decline and differentiated tolerant vs. susceptible cows, suggesting potential as a heat-stress biomarker.	[76]
PI, SM, PG, PE, PC, lyso-PE, lyso-PC, Cer	(1) Extraction system: Folch and Bligh method (2) Chromatography: Kinetex EVO C18 column (2.1 × 100 mm, 1.7 µm; Phenomenex) (3) Detection: TripleTOF 6600	Subclinical intramammary infection by non-aureus staphylococci markedly reshaped the milk lipidome, with 597 out of 2.556 lipids altered > 10-fold. Major shifts involved TAGs and sphingomyelins, revealing inflammation-linked polar lipid changes and potential lipid biomarkers for improved mastitis diagnostics.	[77]
Cer, SM, LPC, PC, PE, PA, PG, PI, PS, LPE, FA	(1) Extraction system: Folch and Bligh methods (2) Chromatography: CORTECS C18 100 × 2.1 mm 2.7 μm column (Waters); XSelect CSH C18 100 × 2.1 mm 2.5 μm column (Waters) (3) Detection: UPLC-HRMS	Quantification of 13 lipid classes across goat, soy, and bovine milk, revealing distinct polar lipid signatures. 14 lipids emerged as authentication biomarkers. Bovine milk was richer in ceramides, TG and DG.	[78]
PA, PC, PE, PG, Cer, SM, HexCer, Hex2Cer	(1) Extraction system: MTBE: MeOH = 5:1 (*v*/*v*) (2) Chromatography: Phenomen Kinetex 1.7u C18 100A column (100 × 2.1 mm) (3) Detection: Triple TOF 6600	Quantitative lipidomics identified 335 lipid species in bovine colostrum vs. mature milk, with 63 significantly altered lipids. Polar lipid shifts (notably in PE, PG, PC, PA, and sphingolipids) highlighted dynamic remodeling of glycerophospholipid and sphingolipid metabolism across lactation.	[79]
PC, Sph species, SM, Cer, HexCer, PE, PS, PG, PA, Hex2Cer, PI	(1) Extraction system: MTBE/MeOH (5:1, *v*/*v*) (2) Chromatography: Phenomen Kinetex 1.7u C18 100 A Column (100 × 2.1 mm) (3) Detection: Triple TOF 6600	Comparative lipidomics across human, bovine, and caprine milk profiled 13 lipid classes, revealing human milk enrichment in TGs with linoleic acid and polar lipids (SM, PLs) containing ARA, DHA, and DGLA. Hundreds of differential lipids were identified, providing biomarkers to guide improved infant formula design.	[80]
PC, PE, SM, Hex1Cer, PG, PI, PS, CerP, Hex2Cer, Sph, PIP, Cer, Lyso-PC, Lyso-PE, PA, Hex3Cer, lyso-PG, lyso-PI, CL, gangliosides.	(1) Extraction system: Modified MTBE protocol (2) Chromatography: Waters Acquity UPLC CSH C18 column (Milford, MA, USA; 1.7 µm, 2.1 mm × 100 mm column) (3) Detection: UHPLC-Q-Exactive Plus-MS	The fermentation process significantly altered the polar lipid profiles of cow milk, involving mainly PC, LPC, PE, LPE, SM, PS, PI, PG, and Cer. Overall, yogurt fermentation decreased the levels of most lipid subclasses.	[81]
Lyso-PA, lyso-PC, lyso-PE, lyso-PG, PA, PC, PE, PE-O, PE-P, PG, PI, PS, Cer, DHCer, Hex1Cer, Hex2Cer, SM.	(1) Extraction system: modified MTBE protocol (2) Chromatography: Phenomenex Kinetex C18 column (2.6 μm, 2.1 mm × 100 mm; Phenomenex Luna NH2 column (3 μm, 2.0 mm × 100 mm) (3) Detection: AB Sciex 6500 + QTRAP MS system	Buffalo colostrum and cow colostrum exhibited more similar lipidomic profiles, but with certain differences, such as higher concentrations of PC (2.78 %), PE (1.52 %), Cer (0.36 %), DHCer (0.03 %), Hex1Cer (0.65 %), and SM (0.86 %) in BC, alongside fewer MG (0.55 %), TG (82.75 %), and LPE (0.19 %) in BC.	[82]
Putatively annotated lipid mass features	(1) Extraction system: adapted from the Folch method (2) Chromatography: ACE Excel 2 Super C18 column (50 × 2.1 mm, 2-μm particle size (3) Detection: Q-Exactive Plus-MS	Significant lipid changes associated with lameness in milk samples from first-lactation dairy cows were revealed using a combination of untargeted LC-MS and ML algorithms LC-MS profiles detected 4235 mass ions. Polar lipids were not discriminant.	[83]
Putatively annotated lipid mass features	(1) Extraction system: modified Folch extraction method (2) Chromatography: Varian C18 column (150 × 4.6 mm); Agilent HILIC column (100 × 2.7 mm) (3) Detection: Agilent 6130 quadrupole LC/MS	LC-MS analysis showed common mass features indicating the presence of mono- and diacyl glycerols and several lysophospholipids among the different types of milk, namely cow, goat, almond, cashew, soy, and coconut.	[84]
Cer, CerG1, CerG2, PG, PI, PS, lyso-PC, lyso-PE, SM, lyso-PI, PC, PE	(1) Extraction system: modified MTBE extraction protocol (2) Chromatography: ACQUITY UPLC CSH C18 column (1.7 μm, 2.1 mm × 100 mm) (3) Detection: Q-TOF-MS	UPLC-QTOF analysis identified 669 lipids (16 classes) in Junggar Bactrian camel vs. cow milk, with clear separation by multivariate models. Seventy differential lipids, especially phospholipids and sphingolipids (PC, PE, PI, PS, SM, Cer), highlight distinct polar lipid signatures supporting authentication and anti-adulteration efforts.	[85]
AHexCAS, AHexCer, AHexCS, AHexSTS, Cer, EtherPC, EtherPE, OxPG, OxPI, PA, PC, PE, PG, PI, PS, SM	(1) Extraction system: butanol/methanol (1:1, *v*/*v*) (2) Chromatography: Hypersil GOLD C18 (2.1 × 100 mm, 3 µm) (3) Detection: Q-Exactive Plus MS	Lipidomic analysis identified 163 significantly differentiated lipids involved in 26 metabolic pathways, primarily related to glycerophospholipid metabolism. Differential lipid expressions in Normande and Holstein raw milk were glycerophospholipids and glycerol lipids.	[86]
EtherPC, EtherPE, EtherPG, EtherPS, lyso-PC, lyso-PE, lyso-PI, Lyso-PS, PA, PC, PE, PI, PS, Cer_NS, Hex2Cer, HexCer_HDS, HexCer_NS, SHexCer, SM.	(1) Extraction system: Methanol, MTBE, Chloroform method (2) Chromatography: Poroshell 120 EC-C18, 3 × 100 mm, 2.7 µm (3) Detection: LC/MS QTOF system	572 molecular species among glycerophospholipids and sphingolipids were detected. Discrimination of plant-based beverages when compared with bovine milk, according to the polar lipid fingerprint.	[87]
PC, SM, PE, PIP, Cer, PS, Hex1Cer, PI, PA, PG, phSM, LPE, CerP, Hex2Cer, LPC, LPS, LPG, LPA, Cer and AcHex derivatives	(1) Extraction system: MTBE-Methanol protocol (2) Chromatography: Waters, ACQUITY UPLC CSH C18, 1.7 µm, 2.1 mm × 100 mm (3) Detection: Q-Exactive Plus MS	2585 lipids detected from the milk samples of the five species in both positive and negative ion modes, categorized into 51 subclasses. PE, SM and PC were the most abundant lipid classes in pig milk samples. TG were the most abundant class in Holstein cow milk.	[88]
PE, PC, PI, SM, Cer, lyso-PC, lyso-PE, PS, PG.	(1) Extraction system: Folch method with some modifications (2) Chromatography: Waters ACQUITY PREMIER CSH C18 Column (1.7 µm, 2.1 × 100 mm) (3) Detection: Q-Exactive MS	17 different polar lipid classes and 353 total lipid species were found in MFGM from cow and camel milk. Particularly, 54 polar lipid species differed significantly in abundance between cow and camel milk.	[28]
Cer, PC, PE, SM	(1) Extraction system: chloroform:methanol (50:50, *v*/*v*) (2) Chromatography: Waters CSH-C18 column (2.1 mm × 100 mm, 1.7 μm particle size) (3) Detection: Shimadzu LC–MS-9030 MS	Smaller MFGs showed higher proportions of polar lipids (including PC, PE and SM) but lower proportions of LMW TGs than larger MFGs in both milks (sheep and cow).	[89]
Hex1Cer, PE, PC, PS; lyso-PC, PC PS, PI, Cer, SM.	(1) Extraction system: MTBE method (2) Chromatography: BEH C8 column (100 m × 2.1 mm × 1.7 μm; Waters) (3) Detection: Q-Exactive hybrid quadrupole Orbitrap MS	Based on the MS/MS spectra, 753 individual lipid species were relatively quantified in cow raw milk. The identified lipid species were mainly fatty acyls, glycerolipids, glycerophospholipids, and sphingolipids. PE, PC, PS, and PI accounted for 23% of the total lipids.	[90]
PI, PE, PS, PC, SM, lyso-PC.	(1) Extraction system: Folch method (2) Chromatography: HILIC column (Waters Atlantis, 2.1 × 150 mm, 5 µm) (3) Detection: maXis UHR-TOF	Significantly different types of phospholipid molecules were screened out in different animal milk samples (cow, goat, donkey, yak). Multivariate statistics revealed that the molecular species of phospholipids in cow and goat milk were more similar than in other animals.	[91]
PC, PS, SM	(1) Extraction system: Isopropanol extraction method (2) Detection: MALDI-TOF-MS	SM and PC identified as potential biomarkers of health status in dairy cows. Both lipids allow for the discrimination of milk from sick animals and also milk from those with stress.	[92]
BisMePA, Cer, dMePE, Hex1Cer, Hex2Cer, LBPA, LdMePE, LPC, LPE, LPI, LPS, PA, PC, PE, PEt, PG, PI, PS, SM, SPH.	(1) Extraction system: MTBE method (2) Chromatography: Accucore C30 column (2.1 × 100 mm with 2.6 μm particles) (3) Detection: Q-Exactive HF-X system	Dietary citrus peel extract enhanced milk yield and antioxidant capacity, increasing unsaturated fatty acids and altering 56 lipid species. Notably, phosphatidylethanolamine and phosphatidylcholine levels rose, indicating improved polar lipid profile and potential to naturally boost milk nutritional quality.	[93]
SM, Cer, HexCer, Hex2Cer, PE, PC, PI, PS, LPE, LPC.	(1) Extraction system: Mixture of methanol, chloroform, water (2) Chromatography: Accucore C18-column (length: 150 mm, internal diameter (ID): 2.1 mm, particle size: 2.6 µm, pore size: 150 Å (3) Detection: Q-Exactive Plus Hybrid Quadrupole Orbitrap MS	Feeding regime strongly shaped the milk lipidome across one year, with 1302 lipids identified. Semi-quantitative analysis showed clear diet- and season-driven changes, including higher odd-chain lipid species in hay-fed cows, underscoring forage-linked shifts in milk polar–lipid composition.	[19]
PC, PE, PS, PA, PG, LPE, LPC.	(1) Extraction system: Modified Folch method (2) Chromatography: CORTECS HILIC Column (2.7 µm, 2.1 mm × 150 mm) (3) Detection: IT-TOF-MS	Processing steps (homogenization, heating, cold storage) reduced bovine milk phospholipids, with boiling and freezing causing the greatest losses. Among 175 phospholipid species, including notable plasmalogens, PC predominated, while heat and storage particularly depleted phosphatidylserine and lysophospholipids, reflecting MFGM disruption.	[20]
Cer, DH-Cer, SM, glycosylated Cer.	(1) Extraction system: methanol-chloroform (3:2) (2) Chromatography: UPLC column (C8, 2.1 × 50 mm, 1.7 µm) (3) Detection: QTRAP 4000 MS	Significant reductions in the concentrations of several milk Cer (e.g., 22:0- and 24:0-Cer) and SM (17:0- and 23:0-SM) in response to high-starch diets by dairy cows.	[94]
SM, LPG, PE, PC, PA, HexCer, PS, Cer, PE-Cer	(1) Extraction system: methyl tert-butyl ether: methanol (50:50, *v*/*v*) (2) Chromatography: Acquity UPLC CSH C18 column (Waters, 100 × 2.1 mm; 1.7 μm) column (3) Detection: IM-QTOF-MS	Significant changes in morning milk of Parmigiano Reggiano involving ceramides and sphingomyelins. Effect of diet and feeding access during the circadian cycle.	[95]

## Data Availability

No new data were created or analyzed in this study.

## Abbreviations

AHexCAS: Acetylated hexosyl ceramide acyl sphingosine; AHexCer: Acetylated hexosyl ceramide; AHexCS: Acetylated hexosyl ceramide sphingosine; AHexSTS: Acetylated hexosyl sulfatide; ARA: Arachidonic acid; BC: Buffalo colostrum; BisMePA: Bis-methyl phosphatidic acid; Cer: Ceramide; Cer-NS: Ceramide non-hydroxy fatty acid–sphingosine; CerP: Ceramide-1-phosphate; DGCer: Dihydroceramide; DG: Diacylglycerol; DHA: Docosahexaenoic acid; DGLA: Dihomo-γ-linolenic acid; ESI/MS: Electrospray ionization–mass spectrometry; FA: Fatty acids; GL: Glycerolipids; GM3: Mono-sialylated ganglioside GM3; GP: Glycerophospholipids; Hex1Cer: Monohexosylceramide; Hex2Cer: Dihexosylceramide; Hex3Cer: Trihexosylceramide; IM: ion mobility; HRMS: High-resolution mass spectrometry; IMS–MS: Ion mobility spectrometry–mass spectrometry; LC–MS: Liquid chromatography–mass spectrometry; LMW: Low molecular weight; LPA: Lysophosphatidic acid; LPC: Lysophosphatidylcholine; LPE: Lysophosphatidylethanolamine; LPG: Lysophosphatidylglycerol; LPI: Lysophosphatidylinositol; LPS: Lysophosphatidylserine; Lyso-PC/LysoPC: Lysophosphatidylcholine; Lyso-PE/LysoPE: Lysophosphatidylethanolamine; ML: Machine learning; MALDI-TOF-MS: Matrix-assisted laser desorption/ionization time-of-flight mass spectrometry; MFG: Milk fat globule; MFGM: Milk fat globule membrane; MG: Monoacylglycerol; MTBE: Methyl tert-butyl ether; NAS: non-*aureus* staphylococci; NMR: Nuclear magnetic resonance; PA: Phosphatidic acid; PC: Phosphatidylcholine; PC-P: Plasmalogen phosphatidylcholine; PE: Phosphatidylethanolamine; PE-P/PE-O: Plasmalogen/ether-linked phosphatidylethanolamine; PG: Phosphatidylglycerol; PI: Phosphatidylinositol; PIP: Phosphatidylinositol phosphate; PLs: Phospholipids; PS: Phosphatidylserine; PUFA: Polyunsaturated fatty acids; QQQ: Triple quadrupole mass spectrometer; QTOF/Q-TOF: Quadrupole time-of-flight mass spectrometer; RP-LC: Reverse-phase liquid chromatography; SCFA/MCFA: Short-chain/medium-chain fatty acids; SFC: Supercritical fluid chromatography; SM: Sphingomyelin; SP: Sphingolipids; ST: Sterol lipids; TAG/TG: Triacylglycerol; THI: Temperature-humidity index; TIC: Total ion current; TIMS/cIMS: Trapped ion mobility spectrometry/cyclic ion mobility spectrometry; UHT: Ultra-high-temperature treatment; UHPLC-MS: Ultra-high-performance liquid chromatography–mass spectrometry; UPLC-HRMS: Ultra-performance liquid chromatography–high-resolution mass spectrometry; UPLC-QTOF: Ultra-performance liquid chromatography-quadrupole time-of-flight.
